# The Research Progress of Bioactive Peptides Derived from Traditional Natural Products in China

**DOI:** 10.3390/molecules28176421

**Published:** 2023-09-03

**Authors:** Yanyan Zhang, Lianghong Liu, Min Zhang, Shani Li, Jini Wu, Qiuju Sun, Shengjun Ma, Wei Cai

**Affiliations:** 1 College of Food Science and Pharmacy, Xinjiang Agricultural University, Urumqi 830052, China; zhangyanyan9527@163.com (Y.Z.); sqj15704438141@163.com (Q.S.); 2 School of Pharmaceutical Sciences, Hunan University of Medicine, Huaihua 418000, China; liulianghongfe@163.com (L.L.); zm18692378021@163.com (M.Z.); 18390218393@163.com (S.L.); hdhd0901@163.com (J.W.)

**Keywords:** bioactive peptides, traditional natural products in China, preparation, isolation, identification, functional classification

## Abstract

Traditional natural products in China have a long history and a vast pharmacological repertoire that has garnered significant attention due to their safety and efficacy in disease prevention and treatment. Among the bioactive components of traditional natural products in China, bioactive peptides (BPs) are specific protein fragments that have beneficial effects on human health. Despite many of the traditional natural products in China ingredients being rich in protein, BPs have not received sufficient attention as a critical factor influencing overall therapeutic efficacy. Therefore, the purpose of this review is to provide a comprehensive summary of the current methodologies for the preparation, isolation, and identification of BPs from traditional natural products in China and to classify the functions of discovered BPs. Insights from this review are expected to facilitate the development of targeted drugs and functional foods derived from traditional natural products in China in the future.

## 1. Introduction

Traditional natural products in China natural products with a rich history and established therapeutic effects [1]. Its active components form the basis for the prevention and treatment of diseases [2]. Currently, the small-molecule bioactive components of traditional natural products in China, such as glycosides, flavonoids, and alkaloids, have been extensively studied [3]. However, macro-molecules, similar to BPs, have received insufficient attention, despite being the highest content of bioactive components in traditional natural products in China and having a wide range of biological activities [4,5]. BPs are sequences composed of 2–20 amino acids that possess therapeutic effects on the body upon ingestion [6]. Depending on their specific therapeutic effects, BPs can be classified as antioxidant peptides, antihypertensive peptides, anti-inflammatory peptides, anti-cancer peptides, etc. [7]. It is worth noting that BPs derived from traditional natural products in China generally do not exhibit biological activity when present in the form of parent proteins and only after the parent protein undergoes enzymatic cleavage by specific enzymes or chemical hydrolysis do the BPs become bioactive [8,9,10]. To date, the largest number of BPs have been obtained from milk proteins, followed by meat, fish, eggs, and cereals, and interest in BPs from other sources is growing [7,11]. Therefore, Over the past few years, there has been a growing interest in exploring BPs in traditional natural products in China and their functional analysis [10]. As a result, numerous functional BPs and chemical structures have been identified in traditional natural products in China, which aligns with the reported therapeutic efficacy of traditional natural products in China [12]. In this paper, we summarize the preparation, isolation, and identification methods of the reported BPs from traditional natural products in China, then discuss the mechanisms of action of BPs such as antioxidant peptides, antihypertensive peptides, anti-inflammatory peptides, and others, and finally briefly discuss their structure-activity relationship, development status, and future trends.

## 2. The Preparation Method of Bioactive Peptides

### 2.1. Enzymatic Hydrolysis

**Figure 1:**
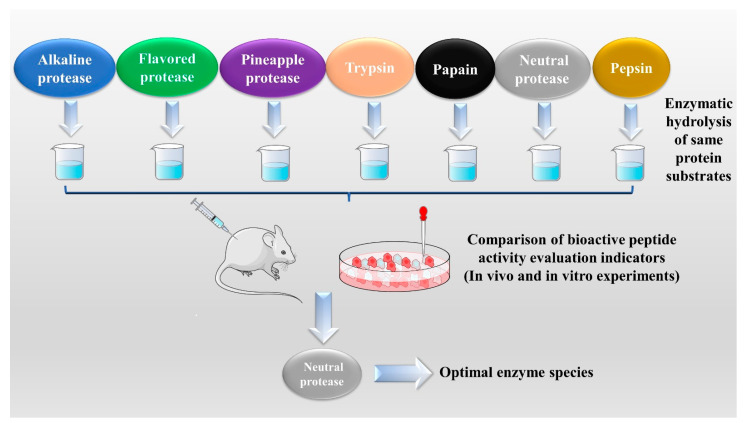
Single enzymatic hydrolysis method.

**Figure 2:**
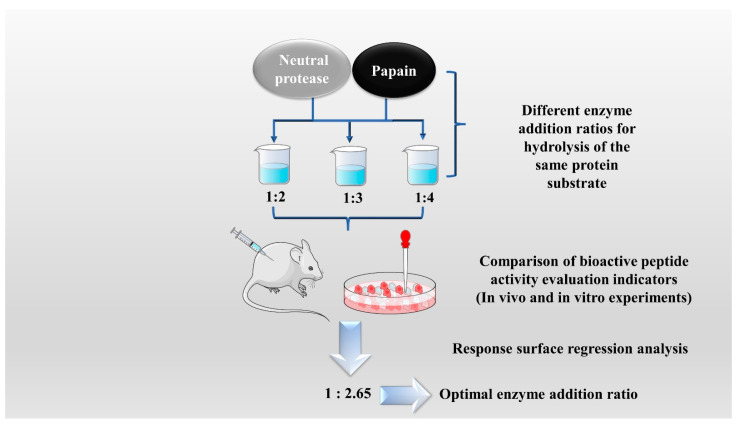
Combinatorial enzymatic hydrolysis method.

**Figure 3:**
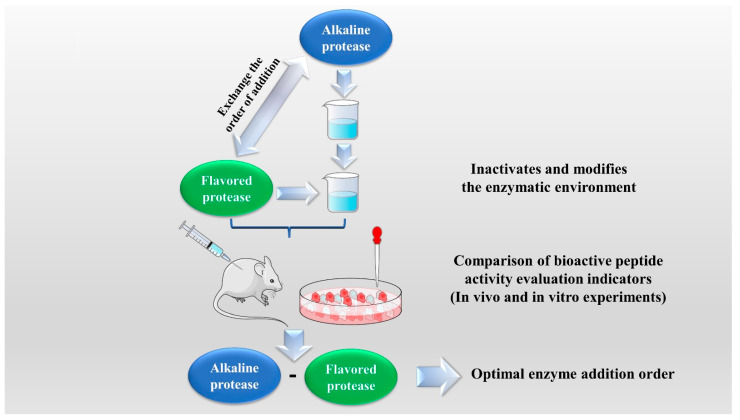
Successive addition of relay enzymatic hydrolysis method.

The enzymatic hydrolysis (EH) of the parent protein from traditional natural products in China is currently the most popular method for obtaining BPs. This is primarily due to the low content of endogenous BPs found in traditional natural products in China, and extracting high-purity endogenous BPs poses certain challenges. Simultaneously, BPs with diverse therapeutic effects obtained by controlling various reaction conditions exhibit greater appeal. EH refers to the hydrolysis of substrate proteins by proteases at a suitable length of time, temperature, pH, and substrate or enzyme concentration, which mainly targets the peptide bonds in proteins to break them down into BPs or amino acid fragments [13]. Therefore, when the conditions of EH or the enzyme species change, it may indirectly impact the efficacy of BPs, firstly, when enzymatic conditions change, factors such as enzyme activity can affect the degree of protein hydrolysis [14]. For example, the length of hydrolysis time is directly related to the degree of hydrolysis, which impacts the molecular weight of BPs and ultimately affects their efficacy in the organism [15]. Secondly, enzymes used in the hydrolysis of protein substrates have specific recognition sites, which are determined by specific amino acids [16]. Changes in these recognition sites can lead to alterations in the amino acid composition of BPs produced by enzymatic hydrolysis, ultimately impacting their efficacy and enzymes currently used for the preparation of BPs from traditional natural products in China, mainly including alkaline protease, flavored protease, pineapple protease, papain, trypsin, neutral protease, and pepsin [17,18]. EH is the most common method for the preparation of BPs from traditional natural products in China. There are two main types of methods: The first is the single EH method, specifically by comparing the efficacy index of BPs obtained by different enzyme hydrolysis, and screening the optimal enzyme species for subsequent experiments. Guo et al. prepared *Hippocampus* (seahorse) peptides using six proteases separately, and the final results showed that *Hippocampus* peptides prepared via papain hydrolysis exhibited elevated antioxidant and anti-fatigue activities. Ghanbari et al. found that among the different proteases tested, alkaline proteases were most effective in generating BPs from sea cucumber (*Actinopyga lecanora*) that possessed the highest levels of ACE-inhibitory and antioxidant activity [19,20] (Figure 1). The second is the complex EH method, which relies on the use of multiple enzyme species. It is worth noting that this method can be further categorized into two subtypes: combinatorial EH and successive addition of relay EH; for the combinatorial EH, Memarpoor-Yazdi et al. incubated the extracted *Zizyphus jujuba* proteins using papain and trypsin and a combination of both, resulting in an ACE inhibition rate of 26.1% (±2.0) for trypsin, which was higher than that of the combined enzymes [21]. Li et al. studied the enzymatic preparation conditions for the combinatorial EH of the BPs of *Lycium barbarum* by neutral protease and papain and obtained the optimal preparation process with a neutral protease to papain addition ratio of 1:2.65 [22] (Figure 2). Successive EH involves a multi-step process in which the first enzyme is allowed to complete its hydrolysis before the EH environment is adjusted and the second enzyme is subsequently added for further hydrolysis, and so on, Bao et al. used dual EH to prepare red deer(*Cervus elaphus*) antler hypoglycemic peptide, and change the order of enzyme species addition for EH, and finally concluded that the EH order of alkaline protease-flavor protease enzymatic products on α-glucosidase glycosidase inhibition rate, protein recovery rate, and hydrolysis degree were 21.11%, 39.12%, and 19.88%, respectively, which were higher than those of the EH order of flavor protease -alkaline protease [23] (Figure 3). Moreover, it was also applied in the preparation of black soybean blood lipid-lowering peptide, iron yam immunomodulatory peptide, and angiotensin-converting enzyme inhibiting peptide from hempseed protein [24,25,26]. As mentioned above, a key step of the EH method is to obtain functional evaluation indexes through repeated in vivo and in vitro functional evaluation experiments in order to screen the best enzyme species or enzymatic conditions, which of course reveals that one of the disadvantages of the EH method is the tedious screening process, besides, the by-products in the mixed peptides and the bitter peptides are also disadvantages, but it is undeniable that the EH method is almost the easiest method to produce consistent hydrolyzed peptides [12,27]. The optimal conditions for the different enzymatic methods obtained by using protein hydrolysis degree or the rest of the functionality indexes as indicators are shown in Table 1.

### 2.2. Solvent Extraction

Solvent extraction is a widely used method for extracting BPs from traditional natural products in China, based on the principle of polar similarity solubility, which can be categorized into aqueous extraction method and organic solvent extraction method depending on the different solvents used, among which aqueous extraction method is the common extraction method of BPs from traditional natural products in China [28,29]. For example, Liu et al. used pure water to extract crushed and sieved *Isatis indigotica* herbs at 4 °C for 24 h to obtain the crude peptides of *Isatis indigotica* [30]. The significant advantage of the aqueous extraction method is the mild reaction conditions, which ensure the stability as well as the safety of the BPs, but this method suffers from a low extraction efficiency and time-consuming procedures [31]. Organic solvent extraction is usually used for proteins or peptides containing aromatic amino acids as well as polar side chains [32]. Liu et al. prepared *Ganoderma lucidum* crude polypeptides using 2500 mL H_2_O/isopropanol (1:15 *w*/*v*) by maceration for 4 h [33]. Li et al. used anhydrous ethanol to extract dried gecko powder to obtain gecko crude polypeptides [34]. Notably, both aqueous and organic solvent extraction methods typically entail the addition of neutral salts such as ammonium sulfate and ammonium acetate to facilitate the precipitation of BPs or proteins [35]. Tie et al. saturated *Achyranthes bidentata* powder aqueous extract solution with ammonium sulfate to 50% saturation, followed by centrifugation, and further saturated with ammonium sulfate to 80% saturation [36]. In addition to the above two methods, adding acid or alkali can also be used for peptide extraction, among which alkali extraction is the most used, and it has been proved that its yield is higher than that of the organic solvent extraction method, mainly because the high pH environment promotes the ionization of acidic and neutral amino acids and disulfide bond breaking, thus improving the solubility of peptides or proteins [31]. However, there are fewer reports on its application to the preparation of BPs from traditional natural products in China, mainly because the high temperature and pH increase required in the process of alkali hydrolysis may exist and difficult to control for the structural damage of BPs leading to their reduced activity or even complete disappearance, so currently for alkali extraction is mainly used in the reuse of agricultural waste such as soybean oil meal in industry and the extraction of animal and plant crude protein, and it is not suitable for the preparation of BPs from traditional natural products in China [37]. The principles as well as the Pros and Cons of the above solvent extraction methods are shown in Table 2.

## 3. The Separation Method of Bioactive Peptides

### 3.1. Membrane Separation

Membrane separation techniques can be categorized into three methods, namely nanofiltration (NF), ultrafiltration (UF), and microfiltration (MF), in ascending order of pore size [38]. Of these, UF is the most widely employed technique for separating peptide mixtures due to its effectiveness and versatility [39]. The main principle of membrane separation is the removal of low molecular weight analytes and impurities through the membrane driven by pressure, while accomplishing the graded separation of peptides of different molecular weights [40]. Ye et al. graded the concentrated supernatant after the EH of sea cucumber (*Stichopus japonicus*) by using a UF membrane bioreactor system with molecular weight cut-off (MWCO) of 5 kDa and collected the fractions with molecular weights less than 5 kDa as sea cucumber peptides, Ding et al. used UF membranes with MWCO of 5 kDa and 10 kDa for the EH of Velvet antler Alcalase and obtained three different molecular weight fractions (>10 kDa, 5~10 kDa, <5 kDa) [41,42]. In addition to UF, MF can be used for impurities removal before the separation of UF components because the filtration pore size is larger than UF [43]. Yuan et al. filtered the supernatant of the aqueous extract of bitter melon (*Momordica Charantia* L. Var. *abbreviata* Ser.) through an MF membrane with a pore size of 0.2 μm, and subsequently, filtered the filtrate through a UF membrane with a MWCO of 10 kDa to obtain two fractions, MC1 (>10 kDa) and MC2 (<10 kDa) [44]. It is worth noting that no reports have been seen on the application of NF to the separation of BPs from traditional natural products in China, mainly because of its MWCO of about 150–1000 Da, which is suitable for completing the separation of small molecules such as oligosaccharides and multivalent ions by dissolution diffusion [45]. Membrane separation techniques have two limitations, Firstly, the consumption of large sample volumes and the potential occurrence of membrane-peptide interactions may result in the loss of target peptides during the separation process, Secondly, the separation efficacy of peptide mixtures at high sample concentrations may be influenced by the “normal distribution” of pore sizes in the membrane, leading to a non-uniform separation behavior [40,46,47,48]. Therefore, membrane separation is only suitable for crude molecular weight separation of peptide mixtures and must be used in cooperation with other separation methods.

### 3.2. Gel Filtration Chromatography

Gel filtration chromatography, commonly referred to as size exclusion chromatography (SEC), separates substances according to their molecular weight by regulating the pore size of stationary phase materials [49]. In this method, the retention time of different components in the peptide mixture varies in the gel column due to differences in molecular weight, leading to a desired separation outcome [50]. The main advantage of SEC is the ease of operation as well as the mildness of the reaction, which is beneficial for the separation of mutability analytes such as BPs and ensures the accuracy of conformational structure characterization [51,52]. The gels packings commonly used for BPs separation are: cross-linked dextran (Sephadex), polyacrylamide (BioGel), cross-linked acryl dextran (Sephacryl), agarose gels (Sepharose), and cross-linked agarose (Sepharose CL), and different types and models of gels can be selected for separation according to the molecular weight level of the sample, among which Sephadex is the most commonly used packings of peptide mixture separation [53]. The mobile phase employed in SEC is typically deionized water, but because of the intrinsic charge properties of peptides, an aqueous mobile phase with a relatively high salt or ionic concentration is often utilized in SEC peptide separations to achieve effective elution and reduce possible changes in peak shape due to secondary interactions with the stationary phase [54]. Wang et al. further separated the membrane-separated fraction F3 of Chinese angelica (*Angelica sinensis*) protein hydrolysate by a Sephadex G-25 gel filtration chromatography column [55]. Mishra et al. extracted the *Ganoderma lucidum* fruiting body (GLF) and *Ganoderma lucidum* mycelium (GLM) powder with Tris-Cl buffer and the supernatant obtained after centrifugation was loaded into Sephadex G-25 to obtain six fractions of GLF 1, GLF 2, and GLF 3 and GLM 1, GLM 2 and GLM 3 [56]. The disadvantage of SEC is the lower peak capacity, which means that a longer column is required to complete the separation, thus affecting the practicability of the method. Therefore, SEC is typically not sufficient for achieving specific separation objectives and is often combined with membrane separation, ion exchange chromatography, or other methods. In protein hydrolysate separation, SEC and/or membrane separation are often utilized in the initial stage to obtain the desired molecular weight or chain length, followed by further characterization to achieve the desired outcome. For example, Zhou et al. used 50 kDa, 10 kDa, 5 kDa, and 1 kDa UF membranes to separate walnut proteolytes into five fractions, which were desalted by a macroporous adsorbent resin and loaded on a Sephadex G-15 chromatographic column to collect the separation obtained at 280 nm walnut ACE inhibitory peptide [57,58]. Notably, SEC generally offers a faster and more efficient separation technique compared to membrane separation, while maintaining a high level of resolution [59].

### 3.3. Ion Exchange Chromatography

Ion exchange chromatography (IEC) is one of the earliest developed chromatographic techniques and is an important tool for the separation, screening, and structural analysis of biomolecules [60,61]. In IEC, the stationary phase possesses ion-exchange properties and interacts reversibly with ions present in the mobile phase, and separation is achieved through the modulation of binding interactions between the stationary phase and the analyte by adjusting the salt concentration or pH of the mobile phase using a gradient elution approach [49,62,63]. IEC is typically classified into strong and weak anion exchange chromatography, which utilize stationary phases featuring quaternary aminoethyl (QAE) and diethylaminoethyl (DEAE) binding groups, respectively, as well as strong and weak cation exchange chromatography which feature propyl sulfonate (SP) and carboxymethyl (CM) binding groups, respectively, these different stationary phases enable the separation of analytes based on their differential interactions with the oppositely charged groups on the stationary phase [64,65]. In addition, in IEC the elution is accomplished by increasing the linear elution salt concentration or buffer pH, but in order to maintain the biological activity of the peptide, it is common to use a phosphate solution gradient elution [66]. Liu et al. uploaded *Ganoderma lucidum* oligopeptide (LZO) LZO-A-3 onto a DEAE-52 cellulose anion exchange column after UF membrane separation and used (NH_4_)_2_SO_4_ solution for gradient elution to obtain a total of six fractions (A-LZO-3-1~A-LZO-3-6) [33]. Zhao et al. obtained (*Lycium barbarum* protein) LBP-A-4 fractions from LBP by UF membrane separation as well as Phenyl Sepharose CL-4B hydrophobic chromatography, and then uploaded onto a DEAE-52 cellulose anion exchange column to obtain 10 fractions (LBP-A-4-1 to 10) after gradient elution with (NH_4_)_2_SO_4_ solution [67]. IEC suffers from two primary drawbacks; firstly, peptides with similar or identical total charges may exhibit poor separation due to their comparable affinity for the stationary phase, necessitating the addition of complementary separation techniques to achieve a satisfactory resolution, secondly, the salt solution employed in gradient elution may introduce unwanted impurity ions, which must be removed via techniques such as desalting using a dialysis bag in order to mitigate potential interference with downstream applications [68].

### 3.4. Reversed-Phase High-Performance Liquid Chromatography

Liquid chromatography is a highly promising technique for separating complex peptide mixtures and proteins in research applications [69]. Of these techniques, RP-HPLC has emerged as the most widely applied approach in proteomics and was first employed for peptide separation back in 1976 [70]. The separation of peptide mixtures by RP-HPLC is mainly based on the differences in polarity and molecular weight, where low molecular weight and low hydrophobic peptides are preferentially eluted in non-polar columns because of the lower interaction forces, resulting in shorter retention times [38,71]. The most commonly used column for peptide separation by RP-HPLC is octadecyl silica, mainly because it is a long-chain alkyl-bonded phase with better hydrophobicity, which is more suitable for biological macromolecules, it is worth noting that when the peptide molecular mass is less than 2 kDa usually use a C18 column with the same pore size as the separation of small molecule mixtures, when the peptide molecular mass is overly large, usually need a column with a pore size range of 30nm~200nm, in addition, the packing materials being studied for peptide and protein separation are Perhydro-26-membered hexaazamacrocycle-based silica (L1lySil), Glycidylmethacrylate (GMA) grafted silica, etc., for the mobile phase, RP-HPLC usually separates peptides by increasing the concentration gradient of the mobile phase of organic solvents (e.g., acetonitrile, methanol or isopropanol), so the selectivity can be improved by changing the composition of the mobile phase and selecting a high purity silica column [72,73,74,75,76]. The use of C18 RP-HPLC column with organic solvent gradient elution has been reported for the separation of velvet antler peptides and *Zizyphus jujuba* peptides, etc. [42,77]. The advantage of RP-HPLC is its remarkable ability to rapidly analyze complex analytes made up of numerous substances, which makes it almost perfectly suited to the task of separating peptides and protein mixtures [65]. On the contrary, RP-HPLC has two disadvantages, the most significant one for peptide mixtures is that the mobile phase consisting of organic solvents and the hydrocarbon bonding in the stationary phase induce changes in the natural conformation of peptides, leading to the loss of their biological activity, and secondly, the steric conformation of peptides may prevent the interaction of hydrophobic amino acids with the stationary phase, leading to the premature elution of peptides [78,79,80].

### 3.5. Multidimensional Chromatographic Separation

It can be concluded from the above summary of various separation methods that each method may be based on only one property of the peptide, and each method has its advantages and disadvantages. For the separation of peptide mixtures, a variety of physical and chemical properties may be involved, such as molecular weight, hydrophobicity, and the number of charges carried, and when the protein is hydrolyzed, the number of peptides to be separated is huge, and exceeds the maximum number of peaks (peak capacity) that most columns can separate in one gradient time, so it is not ideal to use a single method for separation [81,82]. Thus, when multiple properties are used to separate the same peptide mixture, it allows for a mixed-mode separation, which can also be referred to as multidimensional chromatographic separation (MCS), a concept first proposed by Giddings in 1984 [83]. Multidimensional chromatographic separation (MCS) offers two primary advantages over one-dimensional chromatographic separation, first, it enhances separation capacity by independently exploiting multiple separation mechanisms to achieve superior effects, thereby increasing overall efficiency and reducing sample complexity [84]. The second advantage of MCS is the increased peak capacity, which can be optimized by integrating peptide separation techniques that rely on diverse physicochemical properties, specifically, the total peak capacity (N) of MCS should represent the multiplication of the peak capacity (Ni) of each individual separation mode utilized, such as N = N1 × N2 × N3 × … [85]. The most applied system in the separation of BPs is a two-dimensional system consisting of membrane separation and RP-HPLC, such as Lan et al. further separated the New Zealand red deer (*cervus elaphus*) velvet antler antioxidant peptides obtained by UF membrane separation by RP-HPLC to obtain 10 fractions (S1–S10) [86]. In recent years, three-dimensional or even multidimensional liquid chromatographic separation systems with greater separation capacity have been derived and have been applied in peptidomics studies [40,87]. For example, Gao et al. separated ginseng protein crude extracts by UF membrane separation, hydrophobic chromatography, anion exchange chromatography, gel filtration chromatography, and RP-HPLC, and finally purified the five oligopeptide fractions of RSO-1~5 [88]. It should be noted that the incompatibility of mobile phases across different separation modes and the resulting decrease in peak capacity due to incomplete transfer are significant challenges that require attention in future research.

## 4. The Identification of Bioactive Peptides

### 4.1. Identification Based on Database Search

**Figure 4:**
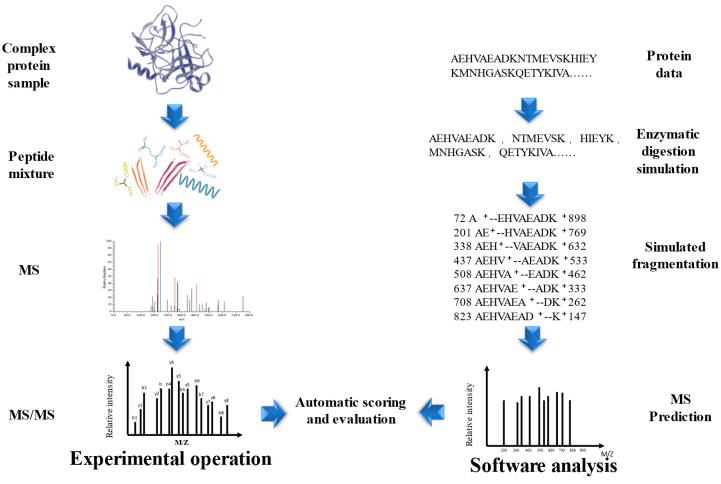
Database search identification peptide method.

The development of MALDI and ESI opened up new possibilities for the integration of multidimensional separation and MS in protein and peptide analysis [89,90]. One established approach is shotgun proteomics, wherein complex protein samples are first fragmented into peptides and then sequenced using LC-MS [91]. Compared to the traditional Edman degradation method, shotgun proteomics offers higher throughput and sensitivity, and has become the preferred bioanalytical tool for proteomics in many laboratories. This method greatly simplifies the sequence identification of peptide mixtures, making it a more convenient option [92]. Database search (DS) is a peptide identification method based on a protein sequence database (PSD), each protein in the PSD is simulated enzymatic cleavage to form peptides after determining the restriction enzyme cutting sites based on the user-specified protease, and simulated fragmentation is performed to obtain the theoretical peptide spectrum, the theoretical peptide spectrum is compared with each experimental MS/MS, the peptide score list is output according to the scoring scheme of the DS tool, and the peptide with the highest score matches has the highest probability of being correct, Thus database based peptide identification is relatively reliable when the complete PSD is known as well as enzyme species [93,94,95,96,97] (Figure 4). Wang et al. used the Orbitrap Elite™ Hybrid Ion Trap-Orbitrap Mass Spectrometer to determine the sequences of sea cucumber peptides and searched the NCBI database, and identified a total of 3961 and 5876 peptide sequences in two fractions, SCP-1 and SCP-2, respectively [98]. Wang et al. used the Uniport database as the basis and searched the raw files of the BPs mass spectra of Colla Corii Asini using the Proteome Discoverer software (Version 2.4.0.305) with the Sequest HT search engine and identified the highest relative content of peptides as ISVPGPMGPSGPR, followed by SGDRGEAGPAGPAGPIGPVGAR and ISVPGPMGPSGPR, the common feature of NCBI and Uniport is the storage of the entire set of annotated or predicted non-redundant protein sequences in the form of FASTA [99,100]. In addition to the above two, there are BIOPEP [101] and SwissProt [102], and it is noteworthy that UniProt contains SwissProt, which is a confidence set for biologically existentially determined proteins [100]. DS peptide identification has several limitations, firstly, it may be challenging to find matches between the mass spectrometry data and the sequences in the database, particularly because some experimental peptides may come from proteins that are not included in the PSD, or they may be variants or products of accidental degradation [103]. For the identification of BPs in traditional natural products in China, the lack of BP sequence information can further complicate the identification process, as BPs are often not well studied [104]. To address these issues, it is necessary to expand the search for relevant PSDs or to explore alternative strategies, such as De novo sequencing or spectral library searching, and it is essential to apply multiple hypothesis testing as well as adjust for the false discovery rate to reduce the chances of false positives [105]. Secondly, non-specific restriction enzyme cutting sites lead to a longer time to calculate results, which results in a lower probability of matching peptide profiles [105].

### 4.2. Peptide Identification Based on De Novo

As mentioned above, when there is a lack of relevant data in the PSD, experimental peptide data may not be able to obtain the correct results after spectral matching by DS. Peptide identification from mass spectrometry data is typically performed using either DS or De novo methods, the former relies on comparing experimental spectra to a protein sequence database, while the latter directly infers peptide sequences based on the mass differences of ion fragmentation peaks acquired by a secondary mass analyzer [93]. Although DS has been a commonly used method for identifying peptides in proteomic research and is generally considered more reliable, recent advancements in high-resolution mass spectrometry instruments have enabled De novo sequencing as a useful alternative method for identifying new peptides [106,107]. In comparison to DS, De novo sequencing can provide more accurate and complete peptide sequence information, especially for complex samples with peptides not included in the database or with multiple modifications. [108]. Therefore, De novo sequencing is becoming a valuable complementary method for peptide identification in proteomics and peptidomics studies. The most obvious advantage of De novo over DS identification is database-independent, secondly, the best current De novo algorithms are orders of magnitude faster than large DS, and finally, De novo sequencing is particularly well-suited for identifying protein sequence variants, splice isoforms, extended peptide sequences, and modified peptides containing non-protein or chemically-modified amino acids [107,109,110]. Zhao et al. sequenced LBP 1~2 by HPLC-ESI-MS and obtained the amino acid sequences of cyclic peptide-(Trp-Glu-Phe-Thr) and cyclic peptide-(Leu-Val-His-Phe), respectively, by manually calculating the M/Z difference [67]. It is worth noting that although De novo sequencing from MS/MS data can be carried out manually, several tools are available that can automate this process [103]. For example, Yingchutraku et al. obtained MS/MS spectra of Ganoderma lucidum water-soluble peptides by Q-TOF mass spectrometry and imported the raw spectra files into Peak X studio 10.0 for automatic De novo sequencing at the highest peptide ion intensity and identified the resulting amino acid sequence as PVRSSNCA [111]. Shih et al. used a Thermo Q—ExactiveTM mass spectrometer to analyze the Cassia obtusifolia Seeds peptides with the highest hypotensive activity and selected the 10 highest intensity ion peaks for secondary mass spectrometry scanning and De novo sequencing using PEAKS Studio to identify the peptide sequence as FHAPWK [112]. In addition, there are software applications such as Bruker Flex Analysis (version 3.3) [77], and De novo explorer [113] for De novo sequencing of BPs in traditional natural products in China. The biggest problem faced by De novo sequencing of peptides is low accuracy, as well as resolution, the most advanced modern sequencing software can only correctly identify peptide sequences in 30–50% of MS, mainly due to incomplete sequences of interfering generated peaks masking sequence-specific fragments and peptides with similar cleavage profiles, Secondly, the requirements for mass spectrometry map quality, are high, mainly because if the peptides are to be accurately De novo sequenced, the fragment ions generated by each peptide bond break need to be observed, and when the MS/MS map quality is poor, it is extremely easy to cause signal loss of fragment ions [97,100,114].

### 4.3. Methods Combination

To improve the accuracy of peptide sequencing, methods have been developed to combine DS with De novo, which is performed by first obtaining a sequence tag of 3–5 amino acids in length by De novo sequencing identification with peptide quality information, and then comparing it with PSD to identify peptides, so this method can also be called sequence tagging method [115,116]. Compared to sequencing peptides using one method alone, firstly, the added specificity of sequence tags can help to reduce the number of hypothetical peptides to be scored during spectrum matching, which can, in turn, help to narrow the scope of downstream DS and reduce analysis time, and secondly, by focusing the analysis on shorter peptide fragments, the accuracy of identifying individual amino acid residues within a peptide sequence can be greatly improved, thereby reducing the potential for false positives or ambiguities in peptide identifications [97,107,117,118]. However, for the combined sequencing methods of BPs of traditional natural products in China, amino acid sequences are mainly obtained by DS alignment and then using De novo sequencing to identify the correctness of sequence results [119], or first sequenced by De novo and then using DS to compare sequence results, there is no relevant report on the study of BPs of traditional natural products in China by sequence tag sequencing [120]. While sequence tags have the potential to improve the specificity and efficiency of peptide sequencing, their use also presents certain limitations and challenges, one key issue is that the amount of information contained in a sequence tag may be relatively limited compared to that of a full peptide sequence, which can hinder the accuracy of identification in some cases, and the risk of peptide misidentification is heightened when errors are made in the identification of the sequence tag itself, which can propagate downstream errors and lead to false positives or other artifacts [121].

## 5. Functional Classification of Bioactive Peptides

The BPs from traditional natural products in China have been classified into various therapeutic categories based on their different effects on the body. These categories include antioxidant peptides, antihypertensive peptides, anti-inflammatory peptides, anticancer peptides, hypoglycemic peptides, hypolipidemic peptides, antimicrobial peptides, anti-fatigue peptides, immune-regulating peptides, muscle synthesis, and performance-enhancing peptides, as well as antifungal peptides. Additionally, the source materials for preparing these different functional BPs from traditional natural products in China may vary. Therefore, Table 3 summarizes the BPs that will be discussed in the corresponding section, providing an overview of the majority of the BPs found in traditional natural products in China thus far.

### 5.1. Antioxidant Peptides

The human body can maintain the antioxidant-oxidant balance via endogenous antioxidant enzymes and non-enzymatic factors to effectively scavenge reactive oxygen species (ROS) under normal conditions, but when this balance is disrupted by external factors such as ultraviolet radiation and excessive exercise, it may lead to various pathological outcomes, so the consumption of antioxidant-rich foods may be an effective strategy to offset the negative impacts of these environmental stressors and safeguard the overall health and well-being of individuals [161,162,163]. Antioxidant peptides are among the most intensively studied BPs from traditional natural products in China, and their activities have been found and confirmed in traditional natural products in China such as ginseng [123,124], deer antler [74,125], Ganoderma lucidum [75,111,122], and Colla Corii Asini [26,119]. The antioxidant pathways of antioxidant peptides mainly include DPPH radical scavenging and ABTS radical scavenging [26,101,111,164,165], liposome oxidation inhibition (LPA) [166], Oxygen Radical Absorbance Capacity (ORAC) [165], and metal ion chelation [111,164], etc. The main factors affecting the efficiency of antioxidant peptides are as follows: firstly, molecular weight and peptide length. In most cases, the number of amino acids of antioxidant peptides is less than 20 and the molecular weight is less than 3k Da, mainly because low molecular weight peptides are easier to reach the active site through biological membrane to scavenge free radicals [167,168,169]. For example, Liu et al. compared the antioxidant activity of antler peptides with different molecular weights by five methods and found that antler peptide III with the smallest molecular weight had the highest antioxidant activity, and the molecular weights were concentrated below 3496 Da [170]. Wang et al. used LC—MS/MS to identify the AsPH-F3 fraction with antioxidant capacity, resulting in 27 of the peptides consisting of 6~19 amino acid residues; another peptide consisting of 26 amino acids [55]. It is worth noting that the antioxidant capacity of a peptide is primarily determined by its specific primary structural features, rather than the presence or absence of individual amino acids as free versus peptide-bound forms, so it is not necessarily the case that smaller molecular weight or shorter peptide segments are intrinsically more potent antioxidants than their larger counterparts [171]. Secondly, the higher the content of hydrophobic amino acids, the stronger their antioxidant capacity is generally considered, mainly because hydrophobic amino acids are more likely to bind to hydrophobic fatty acid radicals and also inhibit the release of hydrogen from lipids by binding to oxygen and delaying the lipid peroxidation chain reaction [172,173]. Liu et al. compared antler peptides I, II, and III and concluded that antler peptide III, which had the highest antioxidant capacity, contained the highest proportion of three hydrophobic amino acids, Leu, Glu, and Gly. [170]. Finally, the N-terminus of antioxidant peptides is generally a hydrophobic amino acid or a tyrosine capable of providing proton-scavenging radicals, as shown by Liu et al. Structural identification of black bean peptides purified by multistage chromatography yielded sequences as Tyr-Asn-Ile and Trp-Asn-Pro, respectively [170]. In addition to this, Cys and Met can scavenge free radicals directly [171].

### 5.2. Anti-Hypertensive Peptides

Hypertension is one of the most common risk factors for the development of cardiovascular disease, and the hypotensive peptides found in plant proteins rely on multiple principles, including ACE inhibition, renin inhibition, proximity to the protective axis of the renin-angiotensin system(RAS), and cholecystokinin (CCK)-related vasodilation, but the hypotensive peptides that have been reported rely mainly on the ACE inhibition mechanism, so they are referred to as ACE inhibitory peptides most of the time [174,175]. ACE inhibition is specifically based on the principle that ACE inhibits vasoconstriction by cleaving the C-terminal dipeptide His-Leu of angiotensin Ι into angiotensin II, and ACE inhibiting peptides inhibit vasoconstriction by competitively or non-competitively inhibiting and thus preventing the binding of ACE to angiotensin I [176,177]. The ACE inhibition efficacy is generally correlated with peptide chain length, because large peptides may have difficulty binding to the active site of ACE, so they are usually peptides with chain lengths of 2-12 amino acids [178]. For example, Memarpoor-Yazdi et al. identified Zizyphus Jujuba fruit peptides fractions F2 and F4 and obtained amino acid sequences Ile-Glu-Arg and Ile-Gly-Lys, respectively, both with higher ACE inhibitory activity than the three longer peptides TNLDWY, RADFY and RVFDGAV purified from Ginkgo biloba seeds [21]. Notably, in some cases, the ACE inhibitory activity of long-chain peptides may be higher than short-chain peptides, mainly because the catalytic site of ACE corresponds to the three hydrophobic amino acids of angiotensin I. Therefore, the activity of ACE inhibitory peptides is also related to the composition of terminal amino acids, of which the three C-terminal amino acids contribute more to the hypotensive effect, mainly Tyr, Pro, Trp, Phe, and Leu [179]. For example, Ma et al. found that the C-terminus of both Ginkgo biloba seeds ACE inhibitory peptides TNLDWY and RADFY contained Tyr, and Shin et al. identified the sequence of Cassia obtusifolia Seeds ACE inhibitory peptide as FHAPWK, whose ACE inhibitory activity was superior to short peptides such as WVYY, WYT, SVYT, and IPAGV due to its C-terminal Pro as well as Trp [112,126].

### 5.3. Anti-Inflammatory Peptides

The inflammatory response of the organism is an important process for external damage and for maintaining the health of the organism, however, when inflammation persists for a long time, the uncontrolled production of multiple biological factors can lead to tissue damage and loss of immune function, resulting in the development of various chronic diseases [180,181]. Currently, inflammatory diseases are mainly treated with small molecule drugs, but their high toxicity as well as a broad range of side effects are very obvious drawbacks in long-term treatment, therefore natural anti-inflammatory peptides with high selectivity, low toxicity, and low dose characteristics are a better choice for the treatment of inflammatory diseases [182,183]. Anti-inflammatory peptides act in the body mainly by regulating the balance of pro-inflammatory factors such as IL-1β, IL-6, IL-12, TNF-α, and anti-inflammatory factors such as IL-10 and TGF-β. The second is to inhibit the expression of nitric oxide synthase (iNOS) and cyclooxygenase (COX-2) metabolic enzymes, thereby reducing the production of inflammatory mediators such as prostaglandins (PGE2) and leukotrienes, and finally to regulate inflammatory signaling pathways such as mitogen-activated protein kinase (MAPK) and nuclear factor-kapa B (NF-κB), thereby regulating the expression of cytokines and inflammatory mediators in signaling pathways [49,184,185]. The main factors affecting the efficacy of anti-inflammatory peptides are the following, firstly, regardless of molecular weight, anti-inflammatory peptides can be absorbed through the intestinal tract intact and modulate inflammatory signaling pathways, but with increasing molecular weight, their effective dose is also increasing [186]. Secondly, when anti-inflammatory peptides carry a positive charge overall, this may make them a factor in regulating inflammatory signaling pathways, thus most anti-inflammatory peptides contain positively charged amino acids, such as His, Arg, Lys, Gao et al. used HPLC-ESI-MS to identify amino acid sequences of five fractions of ginseng oligopeptides-1-5 and determined that all five anti-inflammatory peptides carry His [88,180,187]. Finally, the presence of glutamine, as well as proline, may increase the effect of anti-inflammatory peptides, but their mechanism of action has not been elucidated, while they have not been reported in anti-inflammatory peptides from traditional natural products in China [127,188]. Notably, similar to other BPs, the presence of N-terminal hydrophobic amino acids plays an important role in the activity of anti-inflammatory peptides [129,180].

### 5.4. Anti-Cancer Peptides

**Figure 5:**
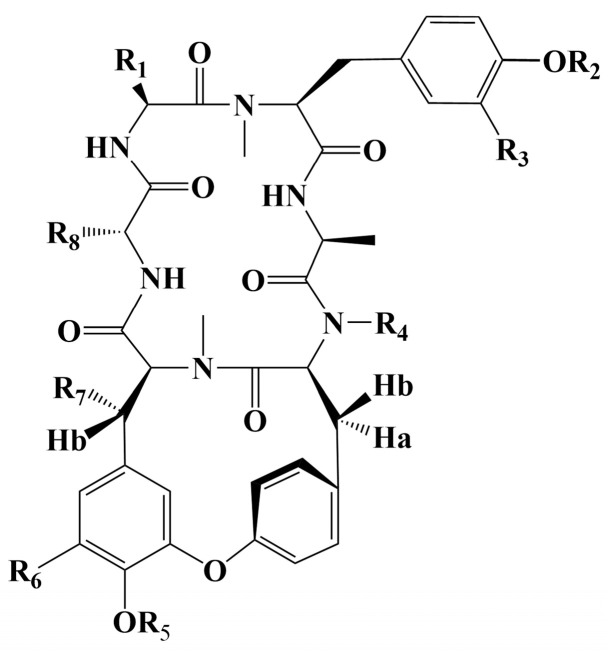
Chemical structure of cyclic peptide from genus Rubia.

**Figure 6:**
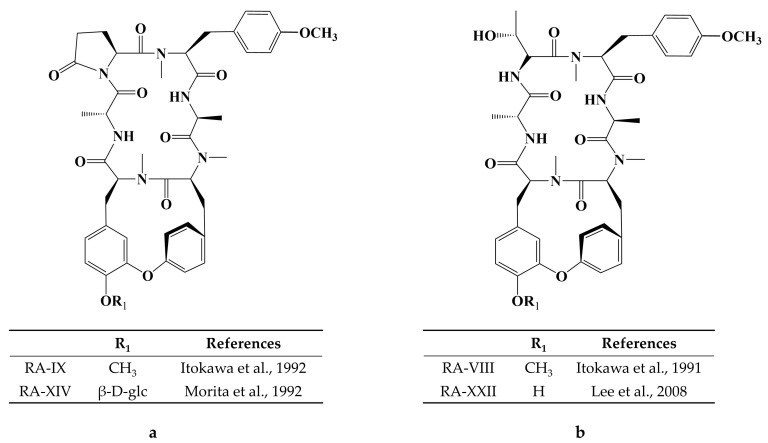
(**a**) Chemical structure of RA-IX, XIV [133,134]; (**b**) Chemical structure of RA-VIII, XXII [132,138].

**Figure 7:**
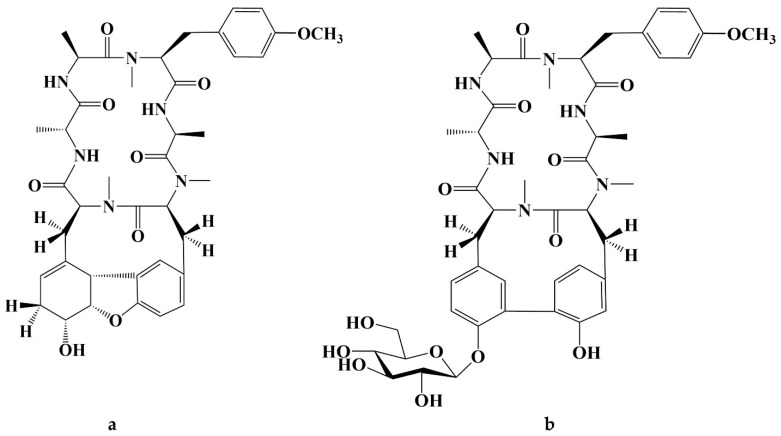
(**a**) Chemical structure of Rubiyunnanin A; (**b**) Chemical structure of Rubiyunnanin B.

Cancer has long been a major problem for people around the world, and the current main treatment for cancer is a combination of surgery, radiotherapy, and chemotherapy leading to modulation of apoptotic pathways and selective induction of apoptosis, but the drawback is the lack of drugs with high specificity, which can easily lead to systemic undesirable effects [189,190,191,192]. Protein-based drugs can accurately induce apoptosis through signaling pathways, while indirectly inhibiting tumor multiplication and possessing lower cytotoxicity, and it is noteworthy that the anti-cancer ability of peptides is now being widely exploited [193,194,195]. The mechanism of anti-cancer peptides is summarized in two main points, which are to destroy the plasma membrane after binding to tumor cells through an electrostatic effect and to induce the release of apoptosis-inducing agents such as cytochrome c from damaged mitochondria [51,196]. The structural characteristics of anti-cancer peptides are mainly as follows, firstly, anti-cancer peptides generally carry positive charges overall, which facilitate electrostatic interactions with anionic lipopolysaccharides in the outer membrane of cancer cells, thus perturbing membrane stability and promoting cell membrane disruption, therefore peptides should carry positively charged amino acids [197,198]. Wang et al. used Discovery Studio 2017 R2 (Dassault Systemes Biovia, San Diego, CA, USA) software to screen Colla Corii Asini peptides with anti-cancer activity based on the value of “-CDOCKER_Energry”, and each of the 10 peptides carrying any one positive charge amino acid at the C-terminus [99]. Then, secondary structure plays an important role in the activity of anti-cancer peptides. The hydrophobic interface formed by α-helix facilitates the diffusion of pore structures generated on the surface of cancer cell membranes, thus inducing apoptosis, and some studies have shown that peptides containing alanine, leucine, glutamine, and lysine are more likely to form α -helix structures [197,199,200]. For example, among the three Agaricus peptides screened by Wang et al. for the best anti-cancer activity, both peptides ANGLTGAK and NGLTGAK contain more than three amino acids that are prone to α-helix formation [201]. It is worth noting that, among the anti-cancer peptides of traditional natural products in China, cyclic peptides are one of the most studied, whose structure is characterized by a closed loop formed by peptide bonds or other chemical bonds between the C-terminus and the N-terminus of the peptide, which has the advantage of mainly good stability in vivo compared to linear peptides, as well as increased binding to cancer cell membrane receptors and cell penetration thus improving cancer therapeutic effects [202,203,204]. The genus Rubia is the main source of anti-cancer cyclic peptides in traditional natural products in China, and 32 cyclic peptides have been identified, mainly including RA-I~V [130], RA-VII [131], RA-VIII [132], RA-IX~X [133], RA-XI~XIV [134], RA-XV~XVI [135], RA-XVII [136], RA-XVIII [137], RA-XIX~XXII [138], RA-XXIII~XXIV [139], RA-XXV~XXVI [140] and Rubiyunnanin A~B [141], Rubiyunnanin C~H [142]. The article summarizes the specific chemical structures in Figure 5, Figure 6 and Figure 7 and Table 4. The anticancer mechanisms of the cyclic peptide RA series derived from the genus Rubia are believed to involve several aspects. Firstly, it is suggested that they might inhibit protein synthesis by binding to the 80S subunit of the eukaryotic cell ribosome, thereby impeding the translocation of aminoacyl-tRNA and peptidyl-tRNA [205,206]. Secondly, they have been found to suppress the NF-kB signaling pathway and inhibit the proliferation, migration, and tube formation of human microvascular endothelial cells [142,206]. It is worth noting that the remarkable stability and enzymatic resistance of natural cyclic peptides have inspired the design and chemical synthesis of cyclic peptides based on their cyclic frameworks [207]. Compared to natural cyclic peptides, artificially synthesized cyclic peptides can be designed with specific sequences to investigate the structure-function relationships of individual amino acids [208]. With the continuous development of cyclic peptide drug research and development, it is necessary to seek more efficient synthetic pathways for cyclization. Currently, the mainstream approach involves the cyclization of linear peptides, synthesized through solid-phase synthesis using artificially designed sequences, by chemoselective peptide or non-peptide bond formation at the peptide segment terminus. In addition to this, enzyme-mediated peptide segment cyclization is also employed [207].

### 5.5. Other Functional Peptides

In addition to the above four functional peptides, there are other functional BPs not introduced, such as hypoglycemic [209], hypolipidemic [148], antibacterial [56], antifatigue [19], and immunomodulatory [152], etc., but these functions BPs are less studied in the field of BPs from traditional natural products in China or the research on the structure-activity relationship is not deep, so we do not dwell on them in this paper and make certain summary in Table 3 above. It is noteworthy that BPs from the same source may have multiple functions, for example, the research team of Professor Li Yong at Peking University evaluated the BPs of Jilin ginseng for multiple activities and found that it possesses six effects simultaneously: delaying alcoholic liver damage, antioxidant, hypolipidemic, hypoglycemic, anti-fatigue, and anti-inflammatory [123,148,150,209,210,211]. The reason could be the functional diversity of different peptides in a mixture of peptides from the same source due to amino acid sequence differences as well as structural differences, secondly, it could be the hydrolysis of the parent protein in different ways due to different preparation processes, and finally, it may be the case that the structural features of a single peptide simultaneously fit the structural requirements of multiple BPs, resulting in a single peptide with multiple functions.

## 6. Discussion

Although research on BPs in the efficacious components of traditional natural products in China is in its preliminary stages, the discovery of BPs has unquestionably enhanced our comprehension of the protein and nutritional attributes of traditional natural products in China as a whole. The development of BPs from traditional natural products in China has been discussed primarily from various perspectives. In terms of preparation, reported methods have included EH and solvent extraction. EH involves hydrolyzing the parent protein of traditional natural products in China to obtain BPs, and it is currently the most commonly used method due to its mild reaction conditions that protect the bioactivity of BPs. However, selecting optimal reaction conditions via efficacy evaluation index is often necessary due to differences in enzymes and complex reaction conditions in EH, which may result in differences in the performance of the final BPs. Yet, this method necessitates cumbersome and complex experimental protocols and lacks studies on exploring the potential BPs of parent proteins. In terms of separation, multidimensional separation methods offer an innovative approach to peptide separation by incorporating and compensating for the respective strengths and weaknesses of individual separation methods. This method makes full use of the physical and chemical properties of BPs to isolate complex peptide mixtures into simpler peptide samples. However, it is important to emphasize that increasing the number of separation dimensions in multidimensional separation methods will not necessarily improve separation efficiency. Rather, it is crucial to ensure a simple and effective interface between different separation dimensions under specific conditions to maximize separation resolution and depth of peptide identification. In terms of identification, there have been attempts to apply both De novo and DS methods. However, for the identification of BPs from traditional natural products in China, these two methods only complement each other’s identification capabilities but do not achieve complete coupling, so the guarantee of accuracy still depends on multiple hypothesis testing and error rate correction. In terms of functional classification and structure-activity relationships, research on traditional natural products in China proteins and BPs is in its preliminary stages, which has resulted in a limited scope of functional and structure-activity relationship studies on some such peptides. However, despite the varied functions stemming from their structural diversity, BPs often exhibit structure features such as smaller molecular weights, shorter peptide chains, and a relatively high content of hydrophobic amino acids.

## 7. Conclusions and Prospect

In conclusion, there are several potential directions for future research and development of BPs in traditional natural products in China. These include optimizing peptide preparation techniques to enhance speed and accuracy, as well as establishing high-resolution multi-dimensional separation systems based on peptide segments. Furthermore, improving traditional natural products in China protein databases, promoting sequence-tagged peptide identification methods, and investigating other functional and conformational relationships, as well as functional mechanisms, of BPs may yield valuable insights. However, it is important to note that ensuring the functional efficacy of BPs from traditional natural products in China in vivo is a key factor for future research and development. This functionality is predicated on the successful delivery of BPs to their corresponding tissues or cells within the organism. Unfortunately, BPs may often lose activity prematurely due to enzyme degradation, structural denaturation, or instability during circulation within the bloodstream, among other factors. Therefore, the targeted delivery of BPs to active sites within the organism can be facilitated through the use of nanocarriers and scaffolds, and this technique has been successfully employed in the development of protein-based anticancer drugs. In the future, BPs from traditional natural products in China should be applied to the development of functional food and peptide drugs, and with the continuous research of structure-activity relationships, it will no longer be difficult to produce the BPs with multiple functions directly by chemical synthesis.

## Figures and Tables

**Table 1:** The optimal conditions for the different enzymatic methods.

Enzymatic Methods	Raw Materials	Types of Enzymes Used	Enzymatic Hydrolysis Conditions	Reference
Single EH	*Ginkgo biloba* seed	Alcalase	pH value: 8.0, temperature: 50 °C, time: 4.5 h, enzyme addition: 3500 U/g.	[17]
	Alfalfa (*Medicago sativa* L.) Leaf	Papain	pH value: 7.5, temperature: 55 °C, time: 4 h, enzyme addition: 3:100 (enzyme/substrate *w*/*w*).	[18]
	Seahorse (*Hippocampus*)	Papain	pH value: 6.0, temperature: 60 °C, time: 40 min, enzyme addition: 2000 U/g.	[19]
	Sea cucumber (*Actinopyga lecanora*)	Alcalase	pH value: 8.0, temperature: 37 °C, time: 8 h, enzyme addition: 1:100 (enzyme/substrate *w*/*w*).	[20]
Combinatorial EH	*Zizyphus jujuba* fruits	Trypsin, papain	pH value: 7.5, temperature: 37 °C, time: 4 h, enzyme addition: 1:50 (enzyme/substrate *w*/*w*).	[21]
	*Lycium barbarum*	Neutrase, papain	pH value: 7.0, temperature: 51 °C, time: 4.3 h, neutral protease-papain addition rate: 1: 2.65.	[22]
Successive EH	Red deer (*Cervus elaphus*) antler	Alcalase- Flavourzyme	Alcalase pH value: 8.0, temperature: 60 °C, time: 3 h, enzyme addition: 5000 U/g, substrate concentration: 12%. Flavourzyme pH value: 6.5, temperature: 45 °C, time: 1 h, enzyme addition: 6000 U/g, substrate concentration: 5%.	[23]
	Black Soybean	Alcalase- Neutrase- Flavourzyme	Alcalase pH value: 8.5, temperature: 60 °C, time: 30 min. Neutrase pH value: 7.0, temperature: 60 °C, time: 60 min. Flavourzyme pH value: 6.0, temperature: 60 °C, time: 30 min.	[24]
	Hempseed (*Cannabis sativa* L.)	Alcalase- Neutrase	Alcalase pH value: 10.0, temperature: 50 °C, time: 4h, enzyme addition: 8000 U/g. Neutrase pH value: 7.0, temperature: 45 °C, time: 4h, enzyme addition: 8000 U/g.	[25]

**Table 2:** The mechanism as well as the Pros and Cons of the solvent extractions.

Methods	Mechanism	Advantage	Disadvantage	Reference
Aqueous extraction	Polar similarity solubility (Polar peptides)	Mild reaction conditions (ensure peptide stability); Simple and convenient operation	Time-consuming; Low extraction efficiency; Introduction of water-soluble impurities	[31]
Organic solvent extraction	Polar similarity solubility (Peptides containing aromatic amino acids and peptides with many nonpolar side chains)	Higher extraction rate (compared to aqueous extraction); Reaction conditions can be controlled to obtain different peptides (e.g., solvent polarity, pH, etc.)	Destruction of essential amino acids such as serine, threonine, and tryptophan; Large solvent usage; Degradation or denaturation of peptides; Peptide toxicity due to organic solvent residues	[31,32,35,37]
Acid or alkali extraction	Disruption of disulfide, hydrogen, and peptide bonds increases peptide solubilization; Acidic and neutral amino acids undergo ionization to increase solubility in a high pH environment			

**Table 3:** Discovered BPs from traditional natural products in China.

Bioactive Peptides’s Functions	Bioactive Peptides Source	References
Antioxidant	*Colla Corii Asini*	[26,119]
	Velvet Antlers	[74]
	*Ganoderma lucidum*	[75,111,122]
	Ginseng (*Panax ginseng* Meyer)	[123,124]
	*Cervus elaphus* velvet antlers	[125]
Anti-hypertensive	*Zizyphus Jujuba* Fruit	[21]
	*Cassia Obtusifolia* Seeds	[112]
	*Ginkgo biloba* seeds	[126]
Anti-inflammatory	walnut	[88]
	Soybean	[127]
	Ginseng	[128]
	Velvet Antler	[129]
	*Lycium barbarum* L.	[67]
Anti-cancer	*Colla Corii Asini*	[99]
	genus *Rubia*	[130,131,132,133,134,135,136,137,138,139,140,141,142]
Hypoglycemic	*Torreya grandis Merrillii*	[143]
	Ginseng	[144]
Hypoglycemic Hypolipidemic	Red Deer Antlers	[145]
Hypolipidemic	Hempseed	[146]
	*Hericium erinaceus*	[147]
	*Eupolyphaga steleophaga*	[113]
	*Panax ginseng*	[148]
Antibacterial	Indian *Ganoderma lucidum*	[56]
	*eupolyphaga sinesis* walker	[149]
Antifatigue	Sea Horse *(Hippocampus)*	[19]
	*Panax ginseng* C. A. Meyer	[150]
Immunomodulatory	Ginseng *(Panax ginseng* Meyer*)*	[151]
	*Colla Corii Asini*	[152]
	Iron yam	[153]
	Ginseng	[154]
	*Panax ginseng*	[155]
	*Isatis indigotica*	[30]
Improve muscle synthesis exercise performance	Purple perilla *(Perilla frutescens* L. Britt.*)* seeds	[156]
Antifungal	*Taraxacum officinale* Wigg. flowers	[157]
	*Trapa natans* fruits	[158]
	*Satureja khuzistanica* leaves	[159]
	Beetle *Blaps rhynchopetera*	[160]

**Table 4:** Chemical structure of cyclic peptide from genus Rubia.

	R_1_	R_2_	R_3_	R_4_	R_5_	R_6_	R_7_	R_8_	References
RA-I	CH_2_OH	CH_3_	H	CH_3_	H	H	H	CH_3_	[130]
RA-II	CH_3_	H	H	CH_3_	CH_3_	H	H	CH_3_	[130]
RA-III	CH_2_OH	CH_3_	H	CH_3_	CH_3_	H	H	CH_3_	[130]
RA-IV	CH_3_	CH_3_	H	CH_3_	CH_3_	H	OH	CH_3_	[130]
RA-V	CH_3_	CH_3_	H	CH_3_	H	H	H	CH_3_	[130]
RA-VII	CH_3_	CH_3_	H	CH_3_	CH_3_	H	H	CH_3_	[131]
RA-X	CH_2_CH_2_COOH	CH_3_	H	CH_3_	CH_3_	H	H	CH_3_	[133]
RA-XI	CH_2_CH_2_COOH	CH_3_	H	CH_3_	H	H	H	CH_3_	[134]
RA-XII	CH_3_	CH_3_	H	CH_3_	β-d-glc	H	H	CH_3_	[134]
RA-XIII	CH_2_CH_2_COOH	CH_3_	H	CH_3_	β-d-glc	H	H	CH_3_	[134]
RA-XV	CH_3_	CH_3_	H	CH_3_	β-d-glc-Ac	H	H	CH_3_	[135]
RA-XVI	CH_3_	CH_3_	H	CH_3_	β-d-glc	H	AcO	CH_3_	[135]
RA-XVII	CH_3_	CH_3_	H	CH_3_	H	H	H	CH_2_CH_3_	[136]
RA-XVIII	CH_3_	CH_3_	H	CH_3_	CH_3_	OH	H	CH_3_	[137]
RA-XIX	i-Pr	CH_3_	H	CH_3_	CH_3_	H	H	CH_3_	[138]
RA-XX	CH_2_CH_3_	CH_3_	H	CH_3_	CH_3_	H	H	CH_3_	[138]
RA-XXI	CH_2_CH_3_	CH_3_	H	CH_3_	H	H	H	CH_3_	[138]
RA-XXIII	CH_2_CH_2_CONH_2_	CH_3_	H	CH_3_	CH_3_	H	H	CH_3_	[139]
RA-XXVI	CH_2_CH_2_CONH_2_	CH_3_	H	CH_3_	H	H	H	CH_3_	[139]
RA-XXV	CH_3_	CH_3_	H	H	CH_3_	H	H	CH_3_	[140]
RA-XXVI	CH_3_	CH_3_	H	H	H	H	H	CH_3_	[140]
Rubiyunnanin C	CH_2_CH_2_COOCH_3_	CH_3_	H	CH_3_	H	H	H	CH_3_	[142]
Rubiyunnanin D	CH_2_CH_2_COOH	H	H	CH_3_	H	H	H	CH_3_	[142]
Rubiyunnanin E	CH_2_CH_2_COOH	H	OH	H	H	H	H	CH_3_	[142]
Rubiyunnanin F	CH_2_CH_2_CONH_2_	CH_3_	H	CH_3_	β-d-glc	H	H	CH_3_	[142]
Rubiyunnanin G	CH_3_	H	H	CH_3_	β-d-glc	H	H	CH_3_	[142]
Rubiyunnanin H	CH_3_	CH_3_	H	CH_3_	β-d-glc	H	H	CH_3_	[142]

## Data Availability

Not applicable.

## Sample Availability

Samples of the compounds are not available from the authors.
